# Chikungunya virus: clinical aspects and treatment - A Review

**DOI:** 10.1590/0074-02760170044

**Published:** 2017-08

**Authors:** Rivaldo V da Cunha, Karen S Trinta

**Affiliations:** 1 Fundação Oswaldo Cruz, Campo Grande, MS, Brasil; 2 Universidade Federal do Mato Grosso do Sul, Faculdade de Medicina, Campo Grande, MS, Brasil; 3 Fundação Oswaldo Cruz, Bio-Manguinhos, Rio de Janeiro, RJ, Brasil

**Keywords:** chikungunya, clinical aspects, treatment, diagnosis, review

## Abstract

Chikungunya is a severe and debilitating disease. Currently, Brazil is experiencing an epidemic caused by three arboviruses, which has changed the way health professionals have diagnosed and treated infected patients. The difficulty of diagnosis and the lack of a protocol for patient treatment, which fits Brazilian health system models, have made it difficult to manage this disease. It is necessary to implement a multidisciplinary network of patient care, in which primary care units play the main role. This review aims to present current information regarding the clinical aspects and treatment of Chikungunya virus infection.

Chikungunya virus (CHIKV) is an arbovirus transmitted to humans by *Aedes* mosquitoes. The virus was first described in 1952 during a febrile illness outbreak in Makonde, a province in southern Tanzania (Robinson, 1955). The word chikungunya comes from the Bantu language of the Makonde ethnic group from Tanzania and Mozambique and refers to the curved position of the patient due to debilitating joint pain (Thiberville et al., 2013). Since its description, in 1952, CHIKV has caused millions of human infections in Africa, the Indian Ocean islands, Asia, Europe, and the Americas (Rodrigues et al., 2016).

Human CHIKV infection is characterised by an intense joint pain of abrupt onset, high fever, and rash. The infection is self-limited and acute symptoms usually resolve within one–two weeks. However, this polyarthralgia is recurrent in 30–40% of infected individuals and may persist for years (Schwartz & Albert, 2010). The joint pain caused by CHIKV infection may be debilitating, which can limit even the simplest daily activities.

The first case of autochthonous CHIKV transmission in Brazil was reported in September 2014, in the state of Amapá (AP). That same month, an outbreak caused by a different genotype occurred in the city of Feira de Santana, state of Bahia (BA) (Rodrigues et al., 2016). From the first notification of CHIKV in Brazil to the time this article was written, 305,585 cases of chikungunya had been reported to the Brazilian Epidemiologic Surveillance System and 40% of Brazilian municipalities had confirmed cases. Estimates drawn from a seroepidemiological study performed in BA after the first epidemic wave indicated that during this outbreak, for each reported case of chikungunya, another 1.94 cases were not reported to the health surveillance system (Cunha et al., 2017). CHIKV infection is thus a serious public health problem that may significantly affect both the economy and the health system of the country.

The present review aims to present the current state of CHIKV infection in Brazil and highlight its clinical aspects and treatment. Some of the information described in this article comes from experience acquired in the field, during a research project that is under development in Feira de Santana, which has been following a cohort of individuals infected with the CHIKV for 18 months.

*CHIKV –* CHIKV is a member of the Togaviridae family, genus Alphavirus, and belongs to the Semliki Forest antigenic complex. Among the members of this antigenic complex are Mayaro, O’Nyong-nyong and Ross River viruses, all of which are capable of causing disease in humans (Cleton et al., 2012).

Chikungunya is a positive-sense single-stranded RNA virus that is approximately 12 kb in length. The genome has two open reading frames (ORFs): the 5´ORF, translated from genomic RNA, encodes the nsP1, nsP2, nsP3, and nsP4 non-structural proteins, and the 3´ORF, translated from subgenomic RNA, encodes a polyprotein that is processed into the structural proteins [capsid (C), envelope (E1 and E2), and two peptides (E3 and 6K)] (Schwartz & Albert, 2010).

The viral particle is spherical, approximately 70 nm in diameter, formed by 240 copies of the capsid protein and surrounded by an envelope composed of a lipid bilayer. Inserted in the envelope are 80 trimer-shaped spikes formed by E1 and E2 glycoproteins (Thiberville et al., 2013).

Distinct CHIKV genotypes have been identified and comprise the East, Central and South African isolates [East–Central–South–African (ECSA)], West Africa isolates (West Africa), and Asian isolates (Asian). The Indian Ocean lineage (IOL) was identified in 2004 as a descendant of the ECSA lineage (Nunes et al., 2015, Tsetsarkin et al., 2007).

Viral replication – Different cells are able to sustain CHIKV infection, including epithelial cells, endothelial cells, primary fibroblasts and monocyte-derived macrophages. CHIKV enters the target cell through receptor-mediated endocytosis. To date, no cellular receptors have been identified. After endosome formation, changes in pH promote conformational changes in the envelope proteins that lead to the fusion of the viral membrane with the endosomal membrane. During this phase, the nucleocapsid is released into the cytoplasm. Viral RNA released into the cytoplasm is translated to form the four proteins of the viral replication complex (nsP1–nsP4), which mediates the synthesis of a negative RNA intermediate that serves as a template for the synthesis of the subgenomic and genomic RNA. Subgenomic RNA is translated into the structural proteins of the capsid, pE2 (precursor of E2 and E3), E1, and 6K. In the cytoplasm, the capsid proteins associate to form the nucleocapsid, which incorporates genomic RNA during the maturation process. The envelope precursor proteins are transported to the endoplasmic reticulum and Golgi complex where they undergo post-transductional modifications to form the E1–E2 heterodimer. These proteins are transported to the host cell membrane to form viral envelope spikes. The virus is then freed from the host cell through the budding process (Lum & Ng, 2015, Schwartz & Albert, 2010).

*Epidemiology –* Arboviruses are transmitted to humans and other animals through the bite of haematophagous arthropods. The five main Arbovirus families that cause diseases in humans and other animals are Bunyaviridae, Togaviridae, Flaviviridae, Reoviridae and Rhabdoviridae (Lopes & Nozawa 2014). Several arbovirus species have already been described to be associated with human infection in Brazil, including Dengue virus, yellow fever virus, Mayaro virus, West Nile virus, Oropouche virus, Saint Louis encephalitis virus, Rocio virus, and Venezuelan equine encephalitis virus (Cleton et al., 2012).

Since the 1952 epidemic in Tanzania, chikungunya outbreaks have been noted in several countries in Africa, Asia, Europe, and the Americas (Rodrigues et al., 2016). During the epidemic wave, which began in 2004, in Africa, several outbreaks were reported in many tropical and subtropical regions (Petersen & Powers, 2016). At the end of 2004, the first cases arose in the Indian Ocean islands, including Comoros, Mayotte, Seychelles, Mauritius, and La Reunion (Schuffenecker et al., 2006). Between 2005 and 2007, during the outbreak that occurred in La Reunion, 266,000 cases were reported, affecting approximately 34% of the island population (LoPresti et al., 2014). In 2006, several cases of CHIKV infection were reported in Europe, reaching several countries, including Italy, France, Switzerland, Germany, Belgium, and England (Powers & Logue, 2007).

The first autochthonous CHIKV infection in the Americas was reported in Saint Martin Island, in 2013 (OPAS, 2013). By 2015, autochthonous cases had already been described in Bolivia, Brazil, Colombia, Ecuador, Paraguay, and Venezuela, with more than 50 countries or territories reporting cases of CHIKV infection (PAHO, 2015).

The first case of autochthonous transmission of the CHIKV in Brazil was reported in September 2014, in AP. The genotyping of the viruses isolated at that time revealed the presence of the Asian genotype. In the same month, an outbreak caused by a different genotype (ECSA) occurred in Feira de Santana (Nunes et al., 2015, Rodrigues et al., 2016). By the end of 2014, 3,657 autochthonous cases of chikungunya had been reported to the Brazilian Ministry of Health (MS, 2015). According to Nunes et al. (2015), the presence of two genotypes in the epidemic that is currently in progress in Brazil suggests that approximately 94% of the Brazilian population is at risk for CHIKV infection.

Brazil is experiencing a triple epidemic caused by the dengue, CHIK and Zika viruses. Brazil is a country of continental dimensions, with a population density of 24.2 inhabitants/km2, and a human development index (HDI) of 0.68 (IBGE, 2016). The prevalence of a hot and humid climate with constant rainfall, which is typical of a tropical region, favours the proliferation of the several vectors associated with the transmission of these arboviruses. Tropical regions are the most affected and threats are associated with rapid climate change, deforestation, population migration, disorderly occupation of urban areas, and precarious sanitary conditions that favour viral amplification and transmission (Lopes & Nozawa, 2014).

CHIKV is transmitted to humans by mosquitoes of the genus *Aedes* spp, particularly *Aedes aegypti*, which is one of the most efficient mosquito vectors for arboviruses. This efficiency is mainly because this genus is highly anthropophilic and lives in close proximity to humans (Coffey et al., 2014).

*Aedes albopictus* is the second-largest transmitter of CHIKV. A mutation associated with an amino acid substitution in the enveloped glycoprotein (E1–A226V) allowed the virus to better adapt to the vector, thus increasing its ability to transmit and disseminate the virus. This finding was observed in the strain of *CHIKV* that circulated during an outbreak in the Indian Ocean islands, referred to as the Indian Ocean lineage (Tsetsarkin et al., 2007).

Other species of mosquitoes, from different parts of the world, have the ability to transmit CHIKV, including *Eretmapodites chrysogaster*, *Culex annulirostris*, *Mansonia uniformis, Anopheles stephensi*, and *Opifex fuscus* (Coffey et al., 2014). Transmission through these vectors is related to their geographical distribution and the types of transmission cycles, whether wild or urban.

In addition to vector transmission, the vertical transmission of CHIKV has been identified. On Reunion Island, 7,509 pregnant women were monitored. Based on positive polymerase chain reaction (PCR) or IgM results, 678 had been exposed to the virus. Of these, 39 exhibited viraemia in the intrapartum period and approximately 49% of their newborns were infected (Gerardin et al. 2008).

The transmission of arboviruses through the blood of patients in the viraemic period may cause a major problem for blood donation in endemic areas. During the epidemic in La Reunion, the French blood service (Etablissement Français du Sang) interrupted blood donations on the island. At the time, an estimated 47 of the 37,750 blood bags donated during the epidemic may have been contaminated if the donations had not been interrupted (Brouard et al., 2008). In a study that analysed the presence of viral RNA and specific IgG antibodies for CHIKV in samples collected before, during, and after the epidemics that occurred in Puerto Rico in 2014, 2.1% of the donations were positive for CHIKV RNA. Epidemiologic studies in this population showed that approximately 25% of blood donors were infected by CHIKV and seroconverted during the epidemics (Simmons et al., 2016).

*Clinical spectrum of CHIKV infection –* After the mosquito bite, the virus enters the skin and the bloodstream. Following the initial replication within dermal fibroblasts, the virus spreads through the bloodstream to the liver, muscles, joints, spleen, lymph nodes, and brain. During the first week of infection, the viral load reaches 109 copies/mL of blood. The viraemic period in the vertebrate host may last two–10 days after infection (Kam et al., 2009, Panning et al., 2008).

As with other viral diseases, CHIKV infection may be asymptomatic or produce a variable spectrum of clinical manifestations, ranging from milder forms to severe and disabling conditions.

Asymptomatic infection – The percentage of asymptomatic infections varies from one epidemic to another, between different age groups, according to the circulating strain and possibly according to the investigative models used to evaluate the prevalence of specific antibodies.

Seroepidemiological surveys conducted in the Comoros and Mayotte islands and in India identified percentages of asymptomatic infections of 14, 28, and 17.5%, respectively (Kumar et al., 2011, Sergon et al., 2007, Sissoko et al., 2008). In contrast, in a 12-month community-based prospective study developed in the Philippines, Yoon et al. (2015) reported 82% asymptomatic infections. A similar result was obtained in another community-based cohort study with children aged between two and 14 years in Managua, Nicaragua, with 58.3% asymptomatic infections (Kuan et al., 2016).

The “natural” history of symptomatic chikungunya infection has been classified into three phases: acute, post-acute, and chronic.

Acute phase – Typically, this phase is considered the first three weeks of the disease, i.e., the first 21 days of clinical manifestations. After an incubation period that ranges from two–four days, infected individuals exhibit high fever, polyarthralgia/polyarthritis and intense myalgia, often accompanied by headache, photophobia, and rash. Polyarthralgia is a result of polyarthritis, which is a characteristic of CHIKV infection and usually quite intense and occasionally disabling. Commonly, the polyarthralgia affects limb joints symmetrically and bilaterally and they are typically swollen. A in figure shows lower limb oedema in a CHIKV-infected patient from a cohort followed during our activities in Feira de Santana. This was one of the most frequently observed clinical manifestations in adults in this cohort during the acute phase.

**Figure:**
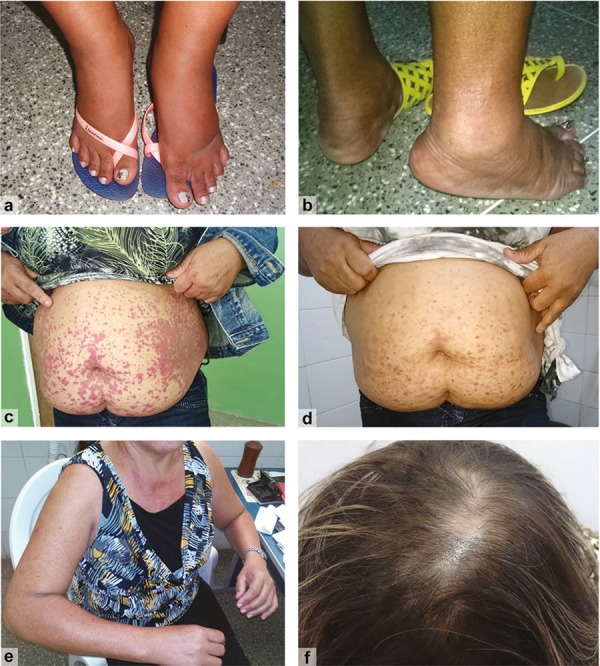
Clinical findings from patients in different phases of the disease. A: oedema of lower limbs, one of the most commonly observed clinical manifestations in adult in the acute phase; B: patient during the acute and post-acute phase presented intense inflammation of the right Achilles tendon, which left as a sequel, the shortening of this tendon in the chronic phase; C: severe cutaneous manifestation in a patient in the post-acute phase, with 80 days of disease, in the fifth episode of lesions onset; D: the same patient referred to in C, after prolonged use of corticosteroids, the lesions disappear, but the hyperchromic spots remained as sequelae; E: restriction of movements in the upper limbs due to the intense inflammatory process in the shoulders and elbows; F: alopecia in a patient in the chronic phase, with approximately 18 months of disease, one of the most frequent clinical findings at this stage, especially among adult women.

Patients often report some stiffness in the distal joints, such as the interphalangeal joints of the hands and feet, ankles, and wrists, particularly upon awakening (Borgherini et al., 2007). Myalgia is more frequent in the arms, forearms, thighs, and calves and may compromise the daily activities of patients, particularly when associated with polyarthralgia/polyarthritis.

Skin manifestations are observed with variable frequency, although they may be exhibited in up to 80% of cases; they involve primarily the face, trunk, and extremities. These manifestations usually appear after the arthralgia and myalgia, between the second and fifth days after the onset of disease, and may persist for an average of two–three days. Macular and maculopapular exanthemas, diffuse erythema, with or without pruritus, and facial oedema are the most common presentations. Other types of cutaneous-mucosal lesions may also be seen in this acute phase, such as vesicles, blisters, exfoliative dermatitis, erythema nodosum, hyperpigmentation, photosensitivity, exacerbation of existing dermatoses, such as psoriasis and ulcers involving the oral mucosa (Borgherini et al., 2007, Seetharam et al., 2012).

General and non-specific clinical manifestations, such as cervical adenopathy, asthenia, chills, nausea, vomiting, and diarrhoea are usually present.

Severe forms of the acute infection have been observed in different age groups, not only in elderly patients. These severe forms usually involve the central nervous system, the respiratory system, and the urinary system. Occasionally, the decompensation of chronic pre-existing diseases, particularly cardiovascular, respiratory, renal, and autoimmune diseases can occur (Renault et al., 2007).

During the acute phase, symptoms that directly affect the daily lives of patients have been observed. On La Reunion Island, a survey conducted with military personnel during the 2006 epidemics evaluated the impact of chikungunya on the quality of life of the interviewees. This study showed that 37.2% of the participants reported disabling fatigue during the acute phase and that 47.3% of the subjects reported substantial or extreme fatigue. According to the same study, 4.6% of the patients reported being extremely depressed and 35.5% reported feeling unmotivated to perform daily activities (Queyriaux et al., 2008).

CHIKV infection is usually self-limited and the acute symptoms usually resolve in one–two weeks. However, polyarthralgia is recurrent in 30–40% of infected individuals and may last for years (Schwartz & Albert, 2010).

Post-acute phase – This phase begins after the 21st day of clinical manifestations and continues for three months (Simon et al., 2015). Only a small proportion of patients remain completely asymptomatic after two–three weeks following the onset of disease. Generally, most patients exhibit only transitory improvements in their clinical condition and relapses occur after a brief “healing” period. Moreover, persistent polyarthralgia or polyarthritis without any change in intensity has been reported by a considerable percentage of patients, which requires analgesic or anti-inflammatory medication to alleviate the pain. What actually appears to occur in these patients is the recrudescence of pre-existing clinical manifestations, albeit of lower intensity.

The percentage of patients with persistent polyarthralgia after the acute phase of CHIKV infection varies according to several factors, such as the genetic susceptibility of the affected populations, cultural differences in how to treat the pain, demographic characteristics, such as age and gender, and the different methodologies used in several existing studies (Sam et al., 2015).

Most studies have indicated that, on average, clinical manifestations persist in 50–90% of patients after the second or third week. This percentage varies according to the age of the affected individuals and is more frequent both in those older than 40 years and in women. In addition to gender and age, factors such as the intensity of the acute-phase manifestations (high fever, arthritis in six or more joints, depression, and high viraemia), a lack of rest during the first days of the disease, and the prior existence of musculoskeletal comorbidities contribute to the persistence of the clinical condition (Simon et al., 2015, Sissoko et al., 2009).

Several studies have described the main clinical manifestations observed during the post-acute phase, which represent the persistence of the initial inflammatory process, including articular and periarticular involvement, whose main clinical manifestations are arthralgia, arthritis (synovitis with or without effusion), tenosynovitis, bursitis, enthesitis, periostitis, and tendinitis, with the risk of tendon rupture. This process may occur in two ways: (i) continuously, with signs and symptoms occurring uninterruptedly, or (ii) with recurrent attacks of intermittent polyarthralgia or polyarthritis, often aggravated by cold (Parola et al., 2007; Simon et al., 2015). E in figure shows a patient, during the post-acute phase, with restriction of movements in the upper limbs due to the intense inflammatory process in the shoulders and elbows.

During the post-acute phase, the decompensation of pre-existing traumatic or degenerative arthropathies, such as osteoarthritis or tendinitis, occasionally calcified, may occur. Additionally, local manifestations, such as reactional oedema and nerve compression syndromes, particularly of the ulnar, medial, and tibial nerves, which produce cubital, carpal, and tarsal tunnel syndromes, respectively, have also been observed. Morning joint stiffness, neuropathic pain, and peripheral vascular phenomena, such as Raynaud syndrome, have also been described (Simon et al., 2015).

Notably, during this phase, a set of non-specific clinical manifestations that are not always associated with chikungunya usually occurs, which may be overlooked by health professionals. The most frequently reported manifestations are chronic fatigue, changes in skin colour (hypo- or hyperchromia), alopecia, decompensated endocrine and metabolic diseases, and decompensation of other pre-existing chronic diseases, such as systemic arterial hypertension, depression, and anxiety (Simon et al., 2015). During our activities in Feira de Santana, we observed several patients with alopecia and changes in skin colour. These findings were more frequent than we expected. F in figure shows an example of a patient who presented extensive hair loss during the chronic phase. C an D in figure show an example of a patient who presented cutaneous manifestations in several regions of the body during the acute phase. After treatment, the lesions disappeared, although hyperchromic spots remained as sequelae.

Chronic phase – The disease is considered chronic when the arthralgia persists for more than three months. The percentage of patients who progress to the chronic phase varies from 40–80% (Chopra et al., 2012, Sissoko et al., 2009) and they may endure clinical manifestations for a few months or even years (Simon et al., 2015).

Significant differences in the spectrum of clinical manifestations and in their intensity and frequency may occur during this phase. As in the acute phase, during the chronic disease, arthralgia and arthritis tend to be bilateral and symmetrical and may be migratory, with pain assuming an intermittent or constant characteristic, possibly accompanied by articular oedema or morning joint stiffness. When oedema occurs, redness and heat are usually not present.

According to a prospective study performed in India with approximately 200 patients, the most frequently observed signs and symptoms during the 10th month of follow-up were joint pain in 46% of patients, fatigue in 13%, and tingling and numbness in the extremities, suggestive of neuritis, in 6%. A subgroup of these patients underwent magnetic resonance imaging exams, which showed joint effusion, bone erosion, bone marrow oedema, synovial thickening, tendinitis, and tenosynovitis (Manimunda et al., 2010).

Among individuals in the chronic phase and regarding the disease progression, three different groups have usually been observed: (i) a larger group in which the disease progresses to cure, spontaneously or after treatment, without lasting sequelae; (ii) a group that is affected by a prolonged persistence of general clinical and/or joint manifestations; and (iii) a group in which the disease tends to become more serious due to the exacerbation of the degenerative or inflammatory process (Simon et al., 2015).

Some studies have revealed that in 80–100% of patients, joint involvement tends to persist for at least six weeks and that, over time, this percentage would decrease to approximately 10% at the end of the second or third year after the acute phase (Waymouth et al., 2013).

Other types of musculoskeletal manifestations may characterise the chronic phase, with the most frequent being tenosynovitis. Typically, two or more tendons are affected, the most common of which are the wrist, finger, and ankle extensors and flexors. Many patients with hypertrophic wrist tenosynovitis complain of nocturnal paraesthesia in the fingers. As in the post-acute phase, cubital, carpal, and tarsal tunnel syndromes have also been described. These syndromes are usually bilateral and accompanied by hypertrophic tenosynovitis (Waymouth et al., 2013). B in figure shows a patient with shortening of the Achilles tendon after an intense inflammatory process occurred during the acute and subacute phases.

The negative impact of CHIKV infection on the patients’ health-related quality of life is quite severe and not limited to the acute disease. Moreover, it may last for several months after clinical recovery (Ramachandran et al., 2012).

Although CHIKV infection is usually benign, increasingly frequent reports have associated chikungunya with decompensation of several pre-existing diseases and an unexpected number of deaths. During the Caribbean and Americas epidemics, between 2013 and 2014, 65 patients were admitted to an intensive care unit with CHIKV infection. Among them, 54 (83%) had pre-existing diseases and 27 were admitted due to an exacerbation of a comorbidity (Crosby et al., 2016). Between 2005 and 2006, during the La Reunion Island epidemics, 237 deaths were associated with CHIKV infection (Schuffenecker et al., 2006). In Colombia, between 2014 and 2015, 58 deaths were reported to the National Colombian Health Institute (Mercado et al., 2016). The number of patients with severe symptoms, a mortality rate from moderate to high, directly or indirectly related to the infection, has changed the understanding of health professionals regarding patient assessment.

*Laboratory diagnosis –* Infections by other Arbovirus in areas that are endemic for dengue are usually incorrectly diagnosed when based solely on clinical and epidemiological data. Many cases of acute febrile diseases have been misdiagnosed and the circulation of other disease-causing arboviruses may be much greater than reported.

The laboratory diagnosis of CHIKV infection is based on viral isolation, viral RNA identification through molecular techniques, such as reverse transcription (RT) and real-time PCR and the detection of IgM and IgG antibodies through serological tests using enzyme linked immunosorbent assays (ELISA) and/or rapid immunochromatographic tests (LoPresti et al., 2014). The choice of a molecular or serological test depends mainly on the time of sample collection.

Viral isolation must be performed using samples collected on or before approximately the eighth day of infection. CHIKV produces cytopathic effect in approximately three days when inoculated in a variety of cell types. The main cell lines used are Vero, BHK-21, and HeLa. The confirmation of CHIKV isolation may be through immunofluorescence or RT-PCR. Although viral isolation is a highly specific method, the time required to obtain results is long. Moreover, a biosafety level 3 (BSL-3) laboratory is required (MS, 2014).

Although techniques based on RT-PCR provide a rapid and sensitive diagnosis, they only permit the detection of viral nucleic acid on or before the seventh day after symptom onset. Regardless, this method is an important tool for the early diagnosis of this infection, particularly in cases of meningoencephalitis and vesiculobullous dermatitis in newborns, for example, in which early diagnosis is essential for successful treatment (MS, 2014).

Available serological tests include ELISA and point-of-care (POC) immunochromatographic tests, which allow IgM detection from the fifth day after symptom onset and IgG detection only a few days later (LoPresti et al., 2014). The advantage of these rapid tests is that because they dispense with a laboratory structure, they may be brought to the field in difficult-to-access locations with no need for refrigeration or equipment and can provide results in 10–20 min.

Notably, cross-reactions with other members of the antigenic complex Semliki Forest, including Mayaro virus (Hassing et al., 2010) have been observed. Therefore, in regions where these viruses circulate, additional tests may be required to confirm the infection.

## Treatment

Pharmacological treatment of pain during the acute phase – The following recommendations were based on a protocol developed by a multiprofessional group for the treatment of pain in chikungunya (Brito et al., 2016). The reference protocol is based on the visual analogue scale (VAS), in which pain intensity varies from 0–10, with 0 signifying the absence of pain and 10 indicating its maximum expression. Occasionally, the stress caused by the disease tends to lead the patients to overstate their pain intensity. For this reason, we recommend complementing VAS with a clinical examination by a physician. Technical guidelines are additionally provided in a document developed by a structured work group at the request of the French Ministry of Health (Simon et al., 2015).

Before beginning treatment during this phase, the physician should heed certain precautions to prevent undesirable reactions:

Ask the patient about any history of allergies or adverse reactions to the medication that will be used (e.g., allergic reactions, arterial hypotension, drowsiness, digestive manifestations).

Investigate the existence of any comorbidity that may cause adverse reactions to the medication used during this treatment stage, such as diabetes, arterial hypertension, glaucoma, renal insufficiency, and cardiomyopathies.

Remember that CHIKV infection may cause decompensation of pre-existing diseases.

Avoid imprecise prescriptions, such as “as needed”.

Avoid non-steroidal anti-inflammatory drugs (NSAIDs) during this phase.

Avoid corticosteroids during this phase, except for specific manifestations, such as neuritis or encephalopathy.

Recall that hydration and absolute rest are crucial components of the integrative approach to the patient.

Address special attention to monitoring the risk of toxicity due to medication, whether due to overdosage, prolonged use, or self-medication.

Mild-intensity pain (VAS from 1 to 3) – The two most commonly used analgesics are dipyrone and paracetamol, which offer quite satisfactory results when they are correctly prescribed. For an adult individual weighing more than 60 kg, dipyrone is recommended at a dosage of 1.0 g every 6 h. Paracetamol may be prescribed at dosages of 500–750 mg every 4–6 h, not exceeding the maximum daily dosage of 4.0 g due to the risk of hepatotoxicity.

Moderate-intensity pain (VAS from 4 to 6) – For pain defined as moderate-intensity, i.e., VAS between 4 and 6, dipyrone and paracetamol should be prescribed together in the same fixed dosages (6/6 h), alternating their administration every 3 h, thereby providing the patient with an analgesic dose of a different product every 3 h.

In cases of allergy to dipyrone, tramadol hydrochloride should be used. However, the use of these two drugs must be considered carefully in pregnant or breastfeeding patients.

When moderate pain does not subside with the use of dipyrone with paracetamol, the 4–Question Neuropathic Pain Diagnostic Questionnaire (DN4) should be applied (Bouhassira et al., 2005).

For patients with severe neuropathic pain, 25 or 50 mg of amitriptyline hydrochloride may be combined with the analgesic being used (dipyrone or paracetamol). Two other medications may also supplement the analgesic: 300 mg of gabapentin twice a day (total of 600 mg/day), with a maximum dosage of 1,200 mg/day or 75 mg of pregabalin twice a day as the starting dose, which may be increased to a maximum dosage of 600 mg/day (300 mg twice a day).

Strict precautions must be followed regarding the use of antidepressants and anticonvulsants. For example, the use of amitriptyline should be avoided in older adults, for whom gabapentin is recommended and must be prescribed in progressive doses. Additionally, the use of amitriptyline is not recommended for patients with either a past or existent history of cardiac arrhythmia.

Severe-intensity pain (VAS from 7 to 10) – To alleviate severe-intensity pain, dipyrone or paracetamol should be combined with an opioid. Opioids may cause nausea and constipation, which may be alleviated easily with the use of routine antiemetics and laxatives.

Among the most frequently used opioid drugs are tramadol hydrochloride, which is typically prescribed at a dosage of 50–100 mg orally every 6 h and 30 mg of codeine combined with paracetamol (500 mg) every 6 h.

Should the pain persist at the same intensity after one week of using an analgesic with an opioid, the DN4 questionnaire must be applied. If confirmed the neuropathic pain one of the previously described options for moderate-intensity pain, with the same characteristic (neuropathic pain), should be used.

If the DN4 questionnaire results exclude the existence of neuropathic pain, the use of corticosteroids or NSAIDs should be considered because, at this point, the disease will have already progressed for two to three weeks. The medications and respective dosages are the same as described for pain in the post-acute phase.

All precautions regarding the use of opioids and corticosteroids must be followed and the exclusion criteria for these classes of drugs must be investigated thoroughly because the prevalence of conditions such as diabetes, hypertensions, glaucoma, and severe cardiomyopathies is quite high among the general population in Brazil.

Pregnant women – Paracetamol is the first option to relieve the pain caused by chikungunya in pregnant women and the dose should not exceed 4 g/day. From the 24th week of gestation onwards, all NSAIDs (including aspirin and topical NSAIDs) are contraindicated due to the risks of foetal renal failure and closure of the ductus arteriosus. Performing a caesarean section to prevent the transmission of CHIKV to the newborn has not been recommended. Conversely, although measures aimed at delaying delivery beyond the viraemic phase have been attempted, positive results have not been consistently achieved (Simon et al., 2015).

Newborns and children – Newborns whose mothers had confirmed viraemia in the period immediately before birth should be placed under neonatal monitoring for five days at the same birthing facility (Simon et al., 2015).

Children with classic chikungunya disease are treated symptomatically as adults, avoiding NSAIDs in infants younger than three months of age or before 10 days of disease progression. Codeine is not recommended for children under 12 years of age and should be reserved for cases refractory to paracetamol.

Treatment of pain in the post-acute phase – The post-acute phase is considered the period between the 22nd day after the onset of disease and the end of the third month. As described, the percentage of patients with sustained clinical manifestations beyond the first three weeks is variable. Prescribing medications for patients in this phase is aimed at relieving joint pain, which is usually a consequence of joint and periarticular inflammatory processes, and therefore, pain relief is conditional on the resolution of the inflammation.

The presence of a neuropathic component to the pain should remain under investigation using the DN4 questionnaire and, if present, the use of antidepressants or anticonvulsants should be recommended. Because the persistence of inflammation and pain magnifies patient suffering, psychological support is essential, and the use of antidepressants, irrespective of neuropathic pain is indispensable.

Mild-intensity pain (VAS from 1 to 3) – For mild-intensity joint pain, i.e., scoring between 1 and 3 by the VAS, NSAIDs are the first therapeutic option. Because many options exist within this drug class, agent selection is often based on the existence of contraindications, which are almost always associated with comorbidities or age. Examples of agents that may be used for the described pain are ibuprofen at a dosage of 400 mg every 8 h, nimesulide (100 mg/day), or meloxicam (7.5–15 mg/day). Satisfactory results have also been obtained with the use of naproxen at 500–750 mg/day. NSAIDs may be used for seven to 10 days, when its use should be reassessed.

When the pain subsides, the medication should be suspended; conversely, should pain complaints persist, corticosteroids should be prescribed at an anti-inflammatory dose. More frequently, prednisone has been used at a dosage of up to 0.5 mg/kg body weight/day, not exceeding 40 mg/day in a single daily dose, administered in the morning. The symptoms usually subside over an average period of approximately three weeks. Should the symptoms subside, the same dosage should be maintained for another three–five days, after which a gradual reduction should be initiated (“weaning”), decreasing by 5 mg every seven days.

Cautions regarding NSAIDs:

Before initiating treatment with an NSAID, any existing comorbidities that may contraindicate its use must be carefully investigated.

In older adults, an evaluation of urea, creatinine, and transaminase levels is recommended before, during, and after NSAID use.

Some comorbidities that contraindicate the use of NSAIDs, when they are decompensated, are very frequent in the adult population (e.g., diabetes, cardiac, renal and hepatic diseases, hypertension, peptic ulcer).

The need to prescribe a gastric protector must always be evaluated.

Moderate- or severe-intensity pain (VAS from 4 to 6 and from 7 to 10) – For moderate- or severe-intensity pain, the first choice is usually corticosteroid therapy. Unless absolute contraindications exist regarding their use, NSAIDs should be used and, if required, given with an opioid, such as tramadol, in the aforementioned doses. Prednisone remains the corticosteroid of choice, at the previously described dose. Conversely, caution is necessary regarding the clinical reassessment of the patient and the appropriate time to initiate the “weaning” process.

A proportion of patients with moderate- or severe-intensity arthralgia usually exhibits recrudescence of the pain condition or perhaps recurrences after short periods of pain relief. When this occurs, the corticosteroid should be reintroduced at the full dose that was previously used. This dose should be maintained for another three or five days after complete symptom resolution and the “weaning” should proceed more gradually, such as by 2.5 mg per week.

For a localised inflammatory process, such as an isolated case of tenosynovitis, bursitis, capsulitis, a tunnel syndrome, or synovitis, which may not respond adequately to oral medication, the recommendation is to use a topical anti-inflammatory or to administer an injection with corticosteroids to the affected region, if necessary. In addition, surgical decompression to relieve recurrent pain from a tunnel syndrome is not recommended due to the risk of poor healing and algodystrophy (Simon et al., 2015).

Pharmacological treatment in the chronic phase – The persistence of clinical manifestations for more than three months from the onset of symptoms is considered a chronic phase. The arthralgia is mild in some of these patients, signifying that the disease is in true regression; in such cases, the joint inflammation is minimal and resolves in a few additional weeks. In contrast, in an appreciable percentage of patients, intense inflammatory manifestations are observed, many of which adequately fulfil the criteria of the American College of Rheumatology to be classified as rheumatoid arthritis.

For mild- to moderate-intensity pain, according to the VAS complemented by the physical examination, the therapeutic recommendations are identical to those previously described. For cases in which the pain intensity is evaluated as strong (VAS from 7 to 10) or for those whose moderate (VAS from 4 to 6), pain is not responding satisfactorily to the therapeutic regimens in use, other drugs must be used. Among these options are the so-called disease-modifying antirheumatic drugs (DMARDS), with methotrexate and hydroxychloroquine. Due to their potential for causing other systemic effects and adverse reactions, these products should be prescribed only by trained professionals; for this reason, their use is not detailed in the current review.

Other approaches to relieving pain and inflammation – Several other interventions can and should be encouraged as additional measures to reduce joint involvement, which contribute to improving patient quality of life.

During the acute phase, rest should be one of the main recommendations. Physical activities tends to aggravate the joint inflammatory process, contributing to local wear and thus to prolonging the clinical condition. Therefore, the need to provide a medical certificate that allows the patient a work exemption during the acute phase should always be evaluated. In the chronic phase, adaptation to another activity that does not require as much effort from the involved joints may often be necessary.

Certain physical and local measures should be encouraged, including (i) the placement of a patch containing an NSAID gel; (ii) joint puncture to drain fluids or to inject corticosteroids; (iii) the (short-duration) use of an orthotic to rest the inflamed joint; and (iv) active and passive movement, which is always made gently to avoid causing pain (Simon et al., 2015).

Antiviral drugs – Several drugs have a recognised effect against CHIKV, when tested in vitro, including ribavirin, interferon alpha, chloroquine, arbidol, favipiravir, and furin inhibitors. However, there is no specific antiviral treatment against the infection (Thiberville et al., 2013).

Vaccine – The first attempts to develop inactivated and attenuated vaccines against CHIKV resulted in products with low immunogenicity and adverse effects such as arthralgia (Harrison et al., 1971, White et al., 1972). Although some options are being evaluated, such as the use of recombinant antigens, viral-like particles, chimeric alphaviruses, electroporated DNA, and CHIKV attenuated by large-scale codon re-encoding, no vaccines are currently available (Thiberville et al., 2013).

As described herein, chikungunya is a disease recently introduced in Brazil, which remains unfamiliar to a significant portion of the population and to health professionals. Its clinical management is complex and often requires laboratory support for diagnostic confirmation of its aetiology, which is not available on a large scale. Conversely, the high temperatures and abundant rainfall, in addition to severe deficiencies in the collection of solid urban waste and an irregular water supply for domestic use, are factors that contribute to the high levels of *Ae. aegypti* infestation that exist in Brazil. For these reasons, it is urgent to organise a multidisciplinary patient care network, in which primary care units will play the leading role.

*Photographs –* Included in this article are photographs of patients at different stages of the disease. All patients had laboratory confirmation of CHIKV infection. The figure shows some of the clinical findings from a cohort of subjects being monitored in Feira de Santana, as part of a research project. The project, entitled *Chikungunya in the municipality of Feira de Santana (Bahia), Brazil: clinical, epidemiological, laboratorial, and quality of life studies*, has been approved by the Ethical Committee from the Feira de Santana State University (1.450.762, 03/14/2016).
